# Dielectric Barrier Discharge based Mercury-free plasma UV-lamp for efficient water disinfection

**DOI:** 10.1038/s41598-017-17455-2

**Published:** 2017-12-12

**Authors:** Ram Prakash, Afaque M. Hossain, U. N. Pal, N. Kumar, K. Khairnar, M. Krishna Mohan

**Affiliations:** 1 CSIR-Central Electronics Engineering Research Institute (CSIR-CEERI), Pilani, 333031 India; 2 Academy of Scientific and Innovative Research, New Delhi, 110001 India; 3 CSIR-National Environmental Engineering Research Institute (CSIR-NEERI), Nagpur, India; 4 Birla Institute of Scientific Research (BISR), Jaipur, India

## Abstract

A structurally simple dielectric barrier discharge based mercury-free plasma UV-light source has been developed for efficient water disinfection. The source comprises of a dielectric barrier discharge arrangement between two co-axial quartz tubes with an optimized gas gap. The outer electrode is an aluminium baked foil tape arranged in a helical form with optimized pitch, while the inner electrode is a hollow aluminium metallic rod, hermetically sealed. Strong bands peaking at wavelengths 172 nm and 253 nm, along with a weak band peaking at wavelength 265 nm have been simultaneously observed due to plasma radiation from the admixture of xenon and iodine gases. The developed UV source has been used for bacterial deactivation studies using an experimental setup that is an equivalent of the conventional house-hold water purifier system. Deactivation studies for five types of bacteria, i.e., *E*. *coli*, *Shigella boydii*, *Vibrio*, *Coliforms* and *Fecal coliform* have been demonstrated with 4 log reductions in less than ten seconds.

## Introduction

Safe drinking water is the basic need of all human beings. Water that is naturally available is generally impure and contaminated with bacteria, viruses, salts and various organic compounds. The sterilization effects of Ultraviolet (UV) radiation is well-known, and low-pressure mercury vapor lamps (with UV radiation at wavelength 254 nm) are generally used for water purification at domestic and industrial levels. There exist different patents that claim UV treatment of water^1–3^. The low-pressure UV lamps contain ~5–50 mg of mercury per lamp whereas low pressure high output lamps contain ~26–150 mg of mercury per lamp^4^. Medium pressure lamps typically contain ~200–400 mg of mercury per lamp^4^. As a consequence, at the end of the lamp life a considerable amount of undesirable toxic waste is generated. Besides, there are other problems associated with mercury based UV-lamps, which include start-up time, filament failure, sleeve breakage, dimensional restrictions and non-reparability.

There have been several efforts towards the improvement of UV light sources^5^ where a pen sized UV lamp powered by a battery and ballast circuitry was used. This lamp is enclosed in a quartz chamber for UV transmission. A UV based apparatus, which produces UV radiation of wavelength (240–280 nm) and also ozone producing wavelength (185 nm) in a gas discharge has been reported^6^. The electrical discharge, especially dielectric barrier discharge (DBD), has been used earlier for ozone synthesis from oxygen^7,8^. A detailed review of use of electrical discharges for water purification has also been published earlier that discusses mainly the ozonization process for water treatment^9^. It is seen that dielectric barrier discharges that operates close to/at atmospheric pressure are efficient sources of UV radiations^10–13^. The wavelength of the UV radiation depends on the type of gas used for creating the discharge, e.g., in plasma display panels (PDP)^14^ millions of micron-sized DBD cells are used to generate UV radiation which in turn is converted to visible radiation through phosphor interaction. Researchers have applied DBD micro-discharge technology for making an energy-efficient and long-lasting backlights in LCD displays^15^ as well as for developing a photo-therapeutic bandage^16^. Another group^17^ developed a cylindrical shaped flexible fluid treatment apparatus with micro-discharge technology. However, the UV radiation from micro-discharge plasmas produced in DBD configuration has not been explored well for water purification. It is to be mentioned that the factors that influence the DBD discharge operation are controlled by many parameter including applied voltage waveforms (i.e., its type, shape, amplitude, rise time, pulse width, frequency, polarity, etc.), geometry of the DBD source, gas composition and pressure (rare gas or mixture with others, etc.), dielectric barrier materials, and types and shapes of electrodes. Hence a critical system optimization is required to develop a water disinfection application using DBD plasma.

In this work, we have engineered an optimized dielectric discharge based mercury-free VUV/UV light source with a novel structural design that produces strong spectral bands peaking at wavelengths 253 nm and 172 nm along with a weak band peaking at wavelength 265 nm, that has been tested on a few representative bacteria to show its usefulness for efficient water sterilization. This invention alleviates most of the problems of contemporary mercury lamps by virtue of its unique design and the composition of active discharge elements. As a result it is able to produce the desired VUV/UV wavelengths for the deactivation of bacteria in water very efficiently –without the use of toxic mercury.

## Experimental Setup

### Source Design

As a first step a mercury-free plasma (MFP)UV-lamp has been developed in a coaxial DBD geometry using quartz tubes [semiconductor grade GE214 USA, (ilmasil® PS by qsil AG, Germany)] with outer tube dimensions 18 mm OD and 15 mm ID and inner tube dimensions 12 mm OD and 9 mm ID, for a lamp length of 195 mm. The quartz tubes have been fused at both the ends using glass blowing technique and a gas gap of 1.5 mm between them has been optimized experimentally, which critically depends upon the discharge conditions including gas pressure, gas type, electrode geometry and applied voltage. Before creating plasma discharge, the developed source was evacuated up to base pressure of ~10^−6^ mbar with the help of standard Rotary and Turbomolecular pumps. The source was then flushed with inert gases (Argon and Xenon) in controlled manner using a mass flow meter (Matheson mass flow transducer series 8272–0453) in the connecting line. This source was again evacuated up to the base pressure which was being monitored by the pressure gauge and the gases were controlled by the needle valves. Research grade xenon with a stated purity of 99.999% was used as a base gas and fractional Iodine was introduced for exciplex plasma formation. The concentration of Iodine has been optimized experimentally by varying its concentration from 5% to 0.0005% of Xe for optimum VUV/UV radiations. The working gas pressure of Xenon was optimized at 150 mbar along with petite iodine concentration of ~0.005% of Xe for maximum VUV/UV radiation.

**Figure 1:**
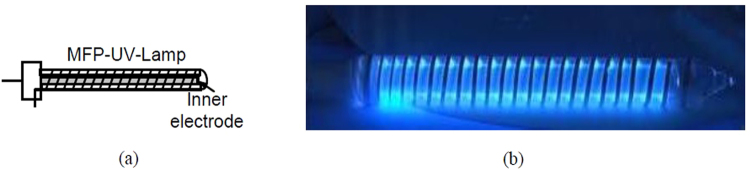
(**a**) Schematic view of MFP-UV-lamp and (**b**) Actual view of MFP-UV-lamp.

The central electrode of the tube is a hollow aluminium pipe of outer diameter 8.8 mm with an aluminium plate welded at the end. This electrode is hermetically sealed with the co-axial quartz tube at one end. The other end of the co-axial quartz tube has also been glass blown and sealed. The central electrode is used for application of high voltage. The outer electrode of the tube is an aluminium metal baked foil tape (with a coating of conductive epoxy on one side), wrapped helically (helix pitch 8 mm, tape width 2 mm) on the surface of the tube. The dimensions of the outer electrode have been optimized experimentally so as to achieve diffused discharge in the lamp. The discharge has been operated between the outer grounded electrode and the inner electrode using variable pulse power supply (pulse voltage 0.5–10 kV; frequency 5–100 kHz and pulse width 1–2 *μ*sec). The schematic and actual view of the developed MFP-UV-lamp are shown in Fig. 1.

### Electrical and Spectroscopic Characterization

**Figure 2:**
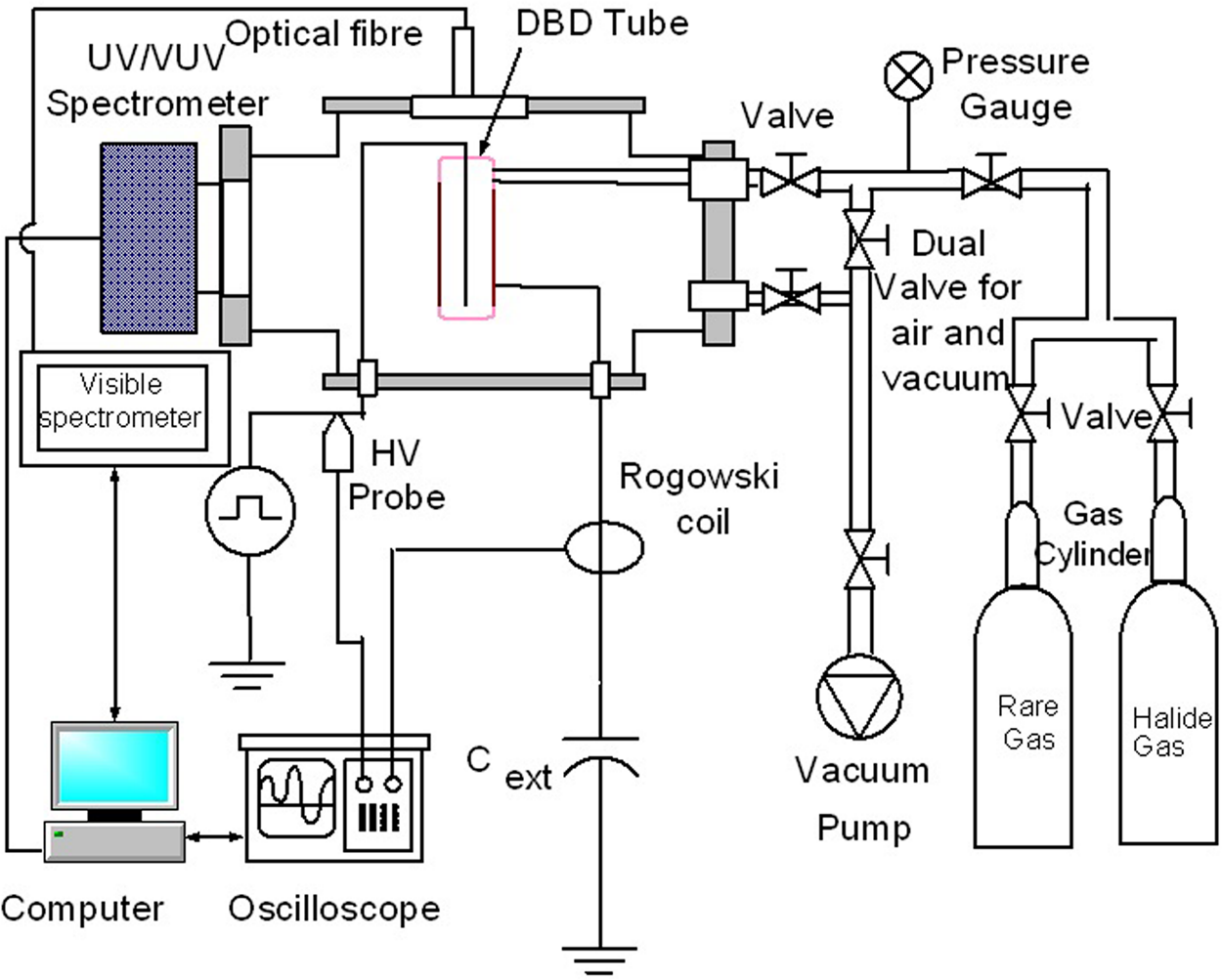
Electrical and spectroscopic characterization setup for MFP-UV-Lamp.

For electrical and spectroscopic characterization, a separate setup was developed as shown in Fig. 2. For electrical characterization, the discharge voltage and current are measured by high voltage probe (Tektronix P6015A) and fast response current transformer (Pearson 110). The oscilloscope (Tektronics DPO 4054) registers the current-voltage characteristics. For the optical characterization of the MFP-UV-lamp, the following instruments were used: Princeton Instruments Monochromator (SP-2500A) with Photo-multiplying Tube (PD 471) (200 nm–400 nm), McPherson VUV Monochromator (Model 234/302) with PMT (McPherson Model 658) (120 nm–200 nm), Lab-sphere (ISS-8P-VA: Integrating Sphere Source), Optical Fiber (LG-455-020-3) 3 meters, 190–1100 nm.

### Test setup for Microbial Analysis

A test chamber equivalent to the 8″ mercury lamp based standard household water container was made. The test chamber was made of Teflon due to its hydrophobic nature. The hydrophobicity of the chamber helps in quick cleaning of the chamber to avoid previous bacterial load while testing deactivation efficiency at repeated time steps. Test chamber for the lamp was made with dimensions 38 mm ID, 48 mm OD and length 205 mm to keep the chamber inner wall 1 cm away from the lamp surface. Following procedure was adopted for the bacterial deactivation efficiency analysis.

1. Already grown bacteria of known quantity were suspended in the sterilized water.
2. Properly mixed bacterial suspension in water was then exposed to the MFP-UV-Lamp by inserting it inside the water in the aforementioned water container. A number of experiments have been performed for different exposure times. Samples of every exposure have been taken and CFU (colony forming units) have been measured using the standard plating technique.
3. There are a number of counting techniques but most rely on the dilution of the sample to reduce the bacterial numbers down to a quantity that can be counted accurately. A series of six dilution samples were prepared and known volume of each dilution was plated on agar plate by standard methods (three samples each for spread plate and pour plate). The serially diluted samples have been enumerated by membrane filtration technique and incubated at 37 °C for 24 hours.
4. Reduction in number of bacteria colonies is then counted after exposure to MFP-UV lamp. The bacterial load is increased serially so as to identify the limit of the deactivation efficiency of the MFP-UV-Lamp. In fact, at sufficiently high seeding concentrations than that of the deactivation efficiency, the UV treatment would result in partial disinfection. In such cases it is not possible to quantify the CFUs effectively after treatment. In those cases, quantification was carried out with the next lower dilution. In every case, the highest countable seeding concentration have been taken into account and the CFU log reduction was calculated from the difference between initial seeding and post-treatment counts.

Tests have been carried out on five different types of gram negative bacteria namely, *Fecal coliform* ((isolated from environmental sources), *Escherichia coli* (ATCC 15597), *Shigella boydii* (ATCC 9207), *Vibrio* (ATCC 9207), *Vibrio* (ATCC17802) and *Coliforms* (isolated from environmental sources).

The developed MFP-UV-lamp has also been tested for turbid water cases in the same test setup. The sterile water was spiked with Kaolin powder for attaining the desired turbidity level. Four turbidity levels namely 0.25 NTU (Nephelometric Turbidity Units), 5 NTU, 10 NTU and 20 NTU were used for the study. Known concentration of bacterial culture were used to spike the turbid water samples.

## Results and Discussions

**Figure 3:**
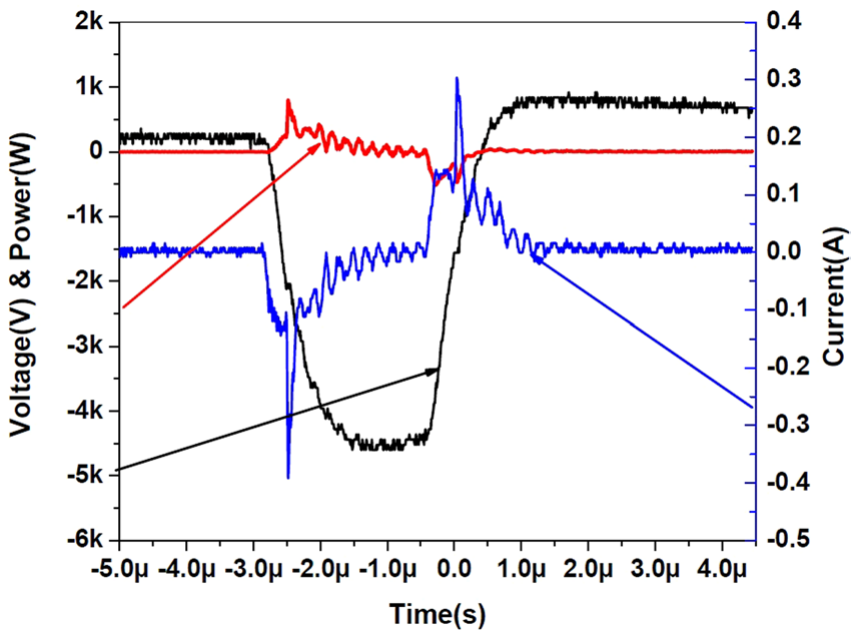
Typical V-I characteristic of the MFP-UV-Lamp.

A typical V-I characteristic of the aforesaid MFP-UV-Lamp is shown in Fig. 3. The energy consumed per pulse has been calculated by integrating power over the pulse duration. This gives a pulse energy of 8.99 × 10^−5^ Joule at the applied frequency of 24.6 kHz with applied pulse voltage of 4.55 kV and pulse duration of 2 *μ*sec. This corresponds to 2.21 W average electrical consumption in the co-axial DBD lamp of outer diameter 18 mm and 195 mm length. Hence the power consumed per unit arc length by this lamp is 0.11 W/cm.

**Figure 4:**
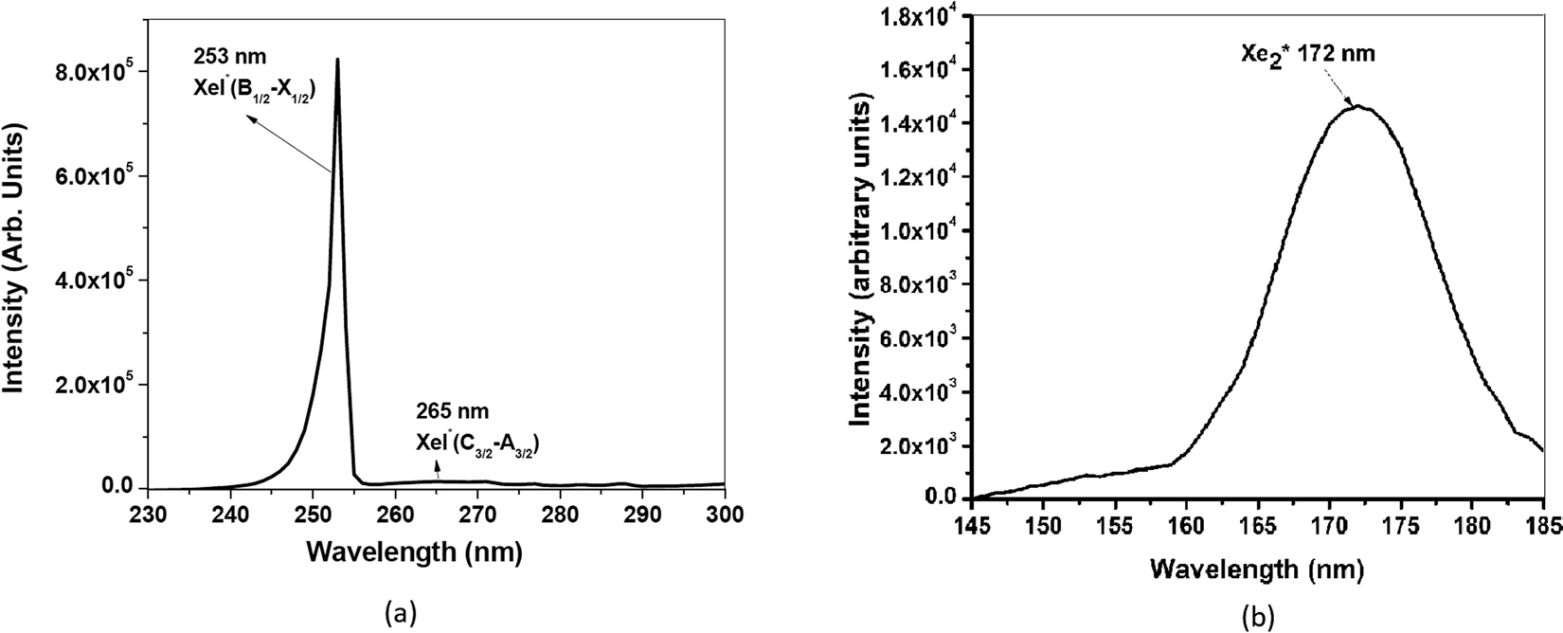
Simultaneously recorded spectra useful for water disinfection in (**a**) UV-C and (**b**) VUV range.

The spectroscopic measurement results from the developed MFP-UV-lamp are shown in Fig. 4. Strong XeI^*^ band (B → X, *λ* = 253 nm) along with strong ${{\rm{Xe}}}_{2}^{\ast }$ band (*λ* = 172 nm) and weak XeI^*^ continuum band (C → A, *λ* = 265 nm) useful for water disinfection have been observed simultaneously. It is to be emphasized that intensity shown above are relative and in arbitrary units. The spectrum recorded in Fig. 4(a) is taken by using Princeton Instruments Monochromator with PMT which is suitable only for 200 nm–400 nm whereas the spectrum recorded in Fig. 4(b) has been taken simultaneously by McPherson VUV Monochromator (suitable for 120 nm–200 nm). The intensity recorded are on different scales in both figures and can not be merged together because the spectrometers chosen are different. For absolute intensity measurements, spectrometer calibration is essential. The calibration curve of the Princeton Instruments Monochromator (SP-2500A) with Photo-multiplier Tube (PD 471) was obtained using Labsphere (ISS-8P-VA: Integrating Sphere Source). The integrating sphere source is often called uniform light source and provides a uniform hemispherical light incident at the reference plane of the light output port and therefore a uniform light output from that port. This effect is created through multiple diffuse light reflections within the hollow sphere and by the sphere shape itself. The absolute intensity measurement of 253 nm wavelength has been carried out for the aforesaid MFP-UV-lamp. It is found to be 4.72 mW/cm^2^ when operated at the applied frequency 24.6 kHz, applied pulse voltage 4.55 kV and pulse duration 2 *μ*sec. A standard commercially available 8″/11 W mercury based UV lamp for 254 nm wavelength has shown 6.52 mW/cm^2^ from this calibration procedure, which was cross verified using G & R Labs radiometer (Model 202) calibrated for 254 nm only. The absolute intensity measurement for 172 nm wavelength cannot be carried out at present at the authors’ laboratory.

**Figure 5:**
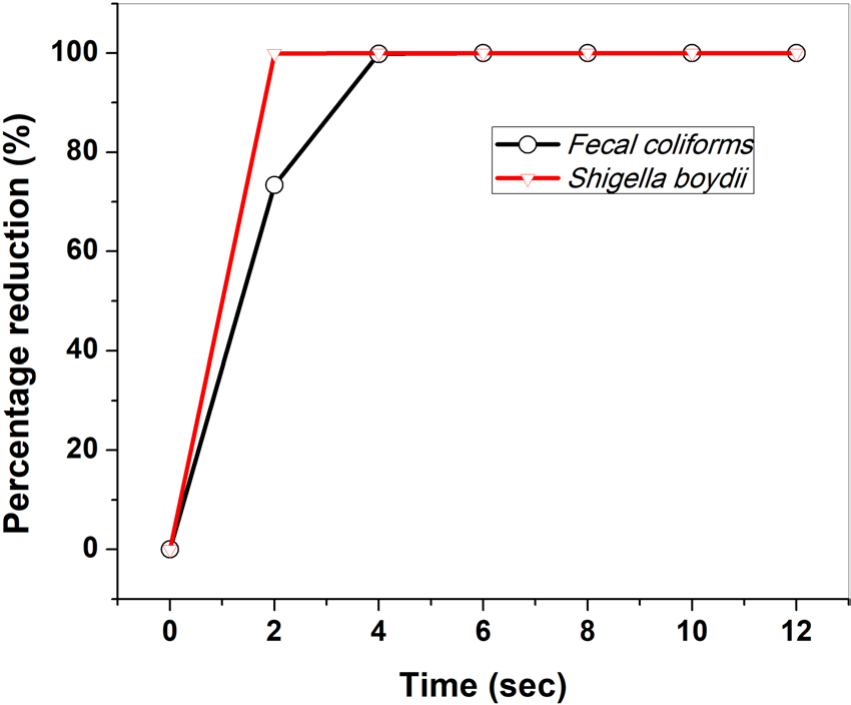
Percentage reduction *Fecal coliform* and *Shigella boydii* at different exposure times.

**Figure 6:**
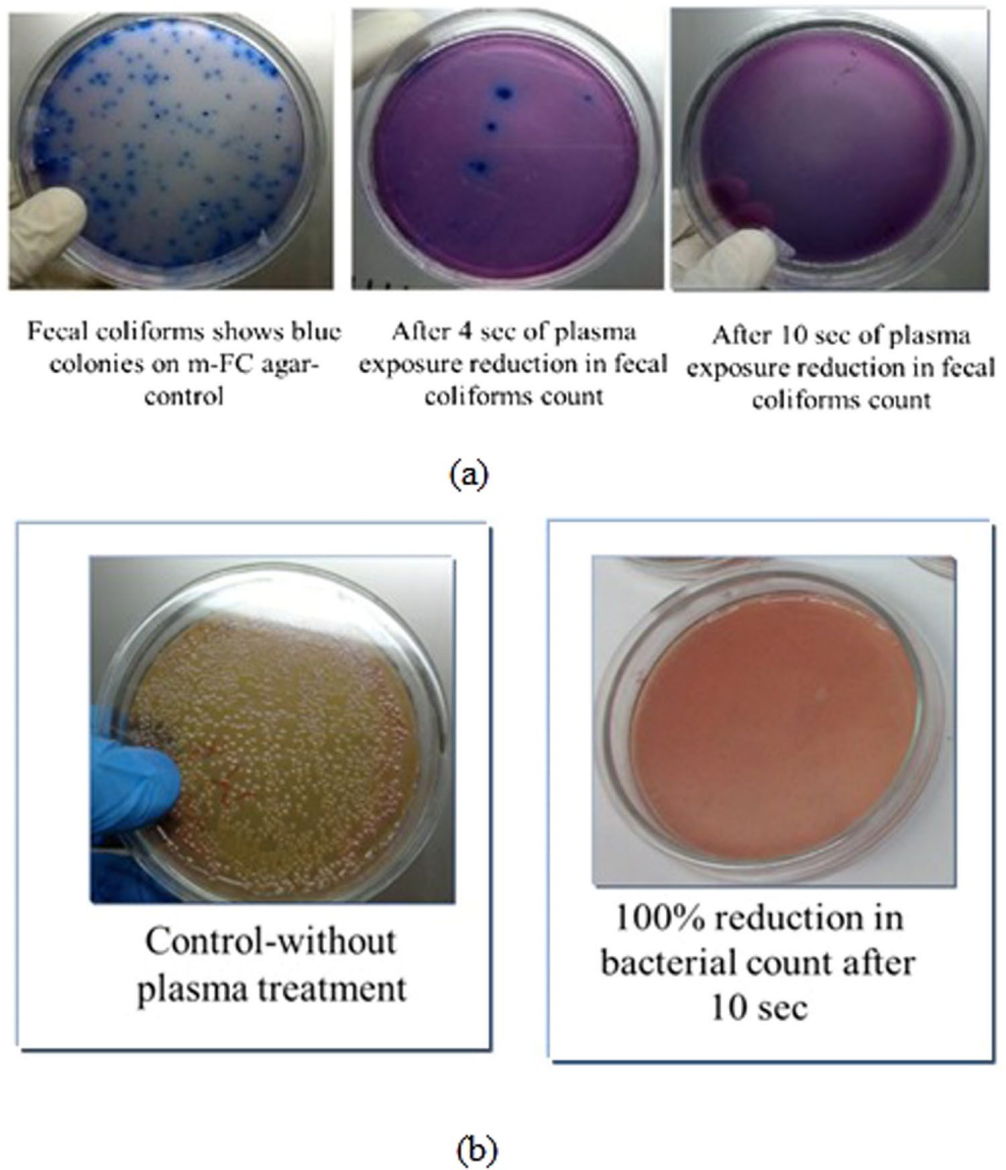
The colony reduction of (**a**) *Fecal coliform* and (**b**) *Shigella boydii* on agar control.

The results of *Fecal coliform* and *Shigella boydii*(ATCC 9207) bacteria deactivation using the developed MFP-UV-lamp with percentage reduction of bacteria is shown in Fig. 5. The colony reduction of *Fecal coliforms* shows blue colonies on m-FC agar control as shown in Fig. 6. It also includes the colony reduction of *Shigella boydii*.

Similar tests have been performed with *Vibrio*, *Coliforms* and *E*. *coli* bacteria deactivations and summary of the bacteria deactivation test results is shown in Table 1. The tests have been performed with *Fecal coliform*, *Shigella boydii*, *Vibrio*, *Coliforms* and *E*.*coli* bacteria deactivations with the average of six samples of control concentrations 7.36 × 10^4^, 5.7 × 10^4^, 2.99 × 10^3^, 4.4 × 10^4^ and 6.2 × 10^4^ CFU/ml, respectively. It also shows the log reduction with 100% deactivation time from their initial concentrations. We have further evaluated the MFP-UV-lamp by using pure 172 nm wavelength to understand its contribution in the bacteria deactivation efficiency (similar lamp size 18 mm O.D. and 195 mm arc length). In this case, discharge has been produced using pure Xenon gas at the same operating parameters and no Iodine admixture has been added. The obtained results for *E*. *coli* bacteria are tabulated below in Table 2 and indicate that wavelength 172 nm in the VUV range is producing some effect though not enough at lower time scales.

**Table 1:** Summary of the five types of Bacteria deactivation.

Bacteria Type	Average CFU/ml	100% deactivation time	Log Reduction
*Fecal coliform*	7.36 × 10^4^	6 sec	4.86
*Shigella boydii*(ATCC 9207)	5.7 × 10^4^	4 sec	4.75
*Vibrio* (ATCC17802V)	2.99 × 10^3^	6 sec	3.47
*Coliforms*	4.4 × 10^4^	8 sec	4.64
*E*. *coli* (ATCC 15597)	6.2 × 10^4^	8 sec	4.79

**Table 2:** Effects of Xe_2_* dimer on *E*. *coli* bacterial deactivation.

Sr. No.	Exposure time	CFU/ml	Log (CFU/ml)	Log reduction	Percentage reduction(%)
1	Control	6.3 × 10^3^	3.8	0	0
2	20 sec	1.58 × 10^3^	3.19	0.61	73.8
3	40 secs	1.4 × 10^3^	3.15	0.65	78.1
4	60 secs	1.22 × 10^3^	3.09	0.71	80.9
5	80 secs	1.18 × 10^3^	3.07	0.73	81.6
6	100 secs	7 × 10^2^	2.84	0.96	89
7	120 secs	6 × 10^2^	2.78	1.02	90.6

VUV photons at wavelength Xe_2_* 172 nm may be useful for ultra-pure water treatment^18^. The photons of this wavelength will not only break organic bonds, but can also generate free radicals. The OH^−^ created may freely react with organic molecules to partially ionize or fully oxidize them to CO_2_ and H_2_O. However, its test is out of scope for the present paper.

The turbidity tests have also been carried out for turbid water at turbidity level 0.25 NTU, 5 NTU, 10 NTU and 20 NTU and obtained turbidity test results have then been compared with the existing mercury based UV-lamp. Exposure times for standard mercury UV lamp and our MFP-UV-Lamp have been compared for water treatment of 1 cm thick water column surrounding the lamp surface and for 4 log reduction. The comparative results of MFP-UV lamp with the existing data^19^ of standard mercury UV lamp are shown in Table 3. The results show that the MPF-UV-lamp has around 200% on-time effciency for deactivation of *E*. *coli* bacteria as compared to standard mercury based UV-lamps.

**Table 3:** On-time efficiency of the MFP-UV-lamp in turbid water.

Turbidity (NTU) Kaoline Powder	Required on-time exposure time for deactivation of *E*. *coli* bacteria (Commercial tube)^19^	Required on-time exposure time for deactivation of *E*. *coli* bacteria (MFP-UV-Lamp)
0.25	12.4 sec	6 sec
5.0	12.8 sec	6 sec
10.0	13.9 sec	8 sec
20.0	15.0 sec	8 sec

**Figure 7:**
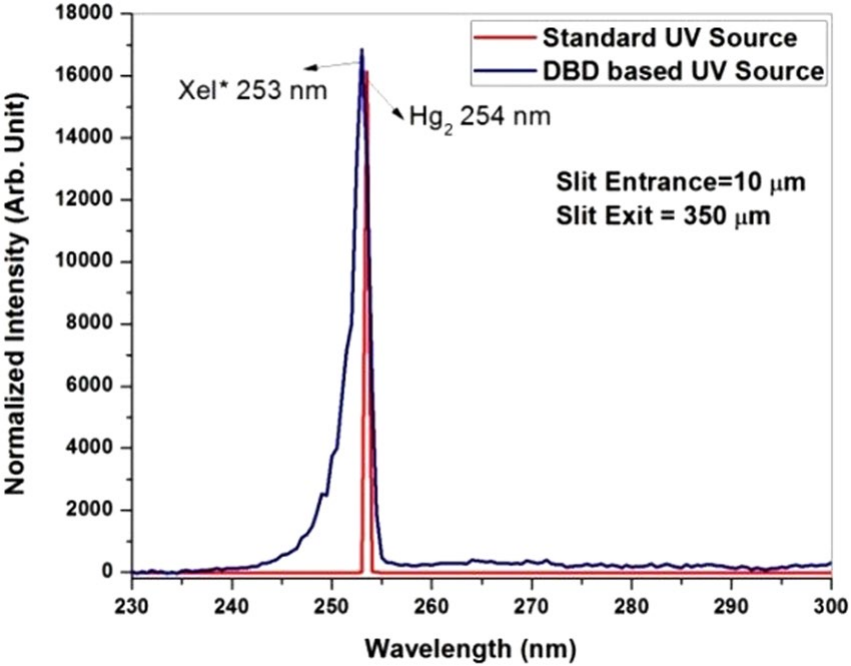
Comparison of UV-C spectra of a standard mercury based UV lamp and MFP-UV lamp.

To understand the higher bacteria deactivation efficiency, a comparative analysis of the spectra of UV-C light from a standard mercury based UV-lamp and MFP-UV-lamp has been carried out as shown in Fig. 7. As illustrated in this figure, the mercury based UV-lamp generates a monochromatic wavelength (line spectrum) due to atomic transition whereas XeI^*^ is a dimer (or molecular) transition and gives a broad band spectrum. In fact, due to non-thermal plasma behavior of the dielectric barrier discharge, it can generate Excimer/Exciplex radiations, which come from diatomic molecules or complexes of molecules that have stable excited electronic states and an unbound or weakly bound ground state. Because the excimer formations are unstable they disintegrate within a few nanoseconds converting their excitation energy to optical radiations having a wide band spectrum^20^. After de-convolution of wide band spectra, multiple wavelengths can be identified in the UV-C range. UV-C radiation used for disinfection is most effective at 264 nm but DNA absorption curve ranges from 200–280 nm. Therefore, more number of transitions due to dimer radiations available in UV-C range in the case of MFP-UV lamp can be the possible causative factor for the increased sterilization efficiency.

## Conclusion

A mercury-free DBD plasma based UV-lamp has been developed using a novel structural design. Strong bands at wavelength 172 nm and 253 nm along with a weak band at wavelength 265 nm have been obtained simultaneously during the plasma discharge. The developed MFP-UV-lamp has been used to expose the bacteria contained water in an equivalent household water purifier system and complete deactivation of five types of bacteria namely, *E*. *coli*, *Shigella boydii*, *Vibrio*, *Coliforms* and *Fecal coliform* with ~4 log reduction has been achieved in less than 10 sec.

The developed MFP-UV-Lamp has also been tested further for water with turbidity level up to 20 NTU by mixing Kaoline powder in the water, and again, its on-time efficiency has been found to be ~200% as compared to standard equivalent mercury based UV-lamp. Besides being mercury-free, the other beneficial features of this MFP-UV-lamp are: Filament-less light source, no end sleeves, negligible start-up time (being a DBD source), scalability in dimensions, broader wavelength coverage due to dimer radiations and medium pressure lamp.
